# Evidence of the medical and economic benefits of implementing hygiene measures by a prevention link physician in trauma surgery: Study protocol for a biphasic multicenter prospective interventional pre-post cohort study using a structured intervention bundle development and tools of behavior change management

**DOI:** 10.1016/j.conctc.2021.100815

**Published:** 2021-07-02

**Authors:** Meike M. Neuwirth, Benedikt Marche, Christiane Kugler, Dominik Bures, Dirk Sauerland, Swetlana Herbrandt, Uwe Ligges, Frauke Mattner, Robin Otchwemah

**Affiliations:** a Institute of Hygiene, Cologne Merheim Medical Center, University Hospital Witten/Herdecke Cologne, Germany, Ostmerheimer Str. 200, 51109 Cologne, Germany; b Witten/Herdecke University, Division of Hygiene and Environmental Medicine, Cologne, Germany, Ostmerheimer Str. 200, 51109 Cologne, Germany; c Division of Trauma and Orthopaedic Surgery, Cologne Merheim Medical Centre, University Hospital Witten/Herdecke Cologne, Ostmerheimer Str. 200, 51109 Cologne, Germany; d Institute for Nursing Sciences, Albert-Ludwigs-Universität Freiburg, Breisacher Straße 153, 79110 Freiburg, Germany; e Witten/Herdecke University, Faculty of Economics, Chair for Institutional Economics and Health Police, Witten, Alfred-Herrhausen-Straße 50, 58448 Witten, Germany; f TU Dortmund, Center for Statistical Consulting and Analysis, Vogelpothsweg 87, 44227 Dortmund, Vogelpothsweg 87, 44227 Dortmund, Germany

**Keywords:** HygArzt, Infection prevention, Implementation of hygiene measures, Prevention link physician, Training program, Hygiene, Trauma surgery/orthopedics, Multicenter prospective interventional cohort study, Wound infection, Surgical site infection

## Abstract

**Introduction:**

The German Commission for Hospital Hygiene and Infection Prevention recommends nominating one authorized medical specialist in every medical department as an infection prevention link physician (PLP). It has been roughly described that a PLP serves as a link between the infection prevention team and the respective clinical departments. No detailed evidence about the contribution made by PLPs to the decrease of infection rates is available in Germany. The “HygArzt” project aims to demonstrate the medical and economic benefits of the implementation of hygiene measures by PLP in trauma surgery/orthopedics.

**Methods:**

A multicenter interventional pre/post cohort study design was chosen. The study will run for a three-year period, including a pre-, post-, and an intervention phase, in four different hospitals, one of which will serve as pilot. A complex intervention containing evidence-based infection control measures will be developed and implemented by a PLP to proof efficacy. After the successful implementation of the preventive measures in the pilot hospital, the concept will be transposed to the three remaining trauma and orthopedic departments to confirm the transferability and generalizability. To enable the PLPs of the non-pilot departments, a subject-specific training program will be developed based on the study results of the pilot hospital and offered to the PLPs.

**Discussion:**

Data are intended to provide evidence that and, if so, to which extent the implementation of specific preventive measures by a medical department-specific PLP is possible and results in a reduction of nosocomial infections in orthopedic surgery and traumatology.

**Contribution to the literature:**

The present study describes a novel complex study design to prove the effectiveness of intervention measures for infection prevention. The study design and newly developed methodological approach could serve as a model for similar studies on infection prevention in the future. For the first time, the presented research project “HygArzt” focuses on the implementation of hygiene measures by an infection prevention link physician (PLP) and investigates whether nosocomial infections, especially surgical site infections, can be reduced by the measures implemented.

**Trial registration:**

German clinical Trials register DRKS-ID:00013,296. Registered on March 5, 2018, https://www.drks.de/drks_web/navigate.do?navigationId=trial.HTML&TRIAL_ID=DRKS00013296.

## 1 Introduction

Nosocomial infections, especially surgical site infections (SSI), pose a high economic burden to national health care systems by leading to prolonged hospital stays, more surgeries, higher treatment costs [1], a reduction of the quality of life of the affected patients, and increased mortality rates. The most prevalent nosocomial infections were SSI, accounting for 22.4%, and resulting in a point prevalence of 1.08% [2]. Therefore, effective infection prevention measures (IPM) are needed. As the number of surgical procedures performed continues to increase [3,4], it is even more important to implement effective SSI prevention measures to minimize the patient's suffering and economic damage [[3], [4], [5]]. Although effective individual measures to reduce SSI are already been implemented, the prevention of infections is often underestimated, such that the number of SSIs has hardly decreased in recent decades [6,7].

Previous studies have shown that antiseptic washing, antibiotic prophylaxis, the use of nasal ointment containing the active ingredient mupirocin as recommended by the KRINKO and WHO, and adherence to hand hygiene are effective individual measures [[8], [9], [10], [11], [12], [13], [14], [15], [16], [17]].

The introduction of IPM bundles appears to be more effective than the introduction of individual measures, as several effective evidence-based measures are combined [18]. However, only a few infection prevention bundles to reduce SSI exist [11,13,14,[18], [19], [20]].

A big challenge is the implementation of IPM [10,21,22]. For a successful implementation, many steps are involved, such as conducting discussions with the superiors, theoretical and practical training of medical staff, and monitoring of the implementation.

The tailoring of IPM to a specific department particularly helps in its implementation. Being part of the department, the infection prevention link physician (PLP) has special knowledge and is therefore in a key position to implement the IPM. According to the German infection protection law and the German Commission for Hospital Hygiene and Infection Prevention (KRINKO) recommendations, a PLP should be in a leading position and must be present in every medical department as a link between the department and the infection prevention team trained in a 40-h prevention course. Their tasks include cooperation with the infection control department for surveillance, transmission, detection, and outbreak management [23]. In addition, the PLP should ensure compliance with hygiene and infection prevention rules, improve hygiene plans and functional procedures, and participate in trainings and further education on hygiene topics. The PLP's core tasks are the promotion of interdisciplinary cooperation in infection prevention and the implementation of IPM. She or he should see her- or himself as an intermediary between the departments [23]. Although the effectiveness of the implementation of IPM in hospital departments by nursing staff was described for infection control link nurses [24,25], no study has investigated the effectiveness of PLP to date. Our study has as aim to evaluate the effects of PLP to optimize medical processes and reduce the number of nosocomial infections. The associated medical and economic benefits as well as the implementation of IPM by PLP in trauma surgery/orthopedics should be evaluated.

## 2 Methods

### 2.1 Aims and objectives

The planned multicenter prospective interventional cohort study would evaluate whether a PLP is able to implement an infection control bundle, which should be developed in collaboration with the infection control department.

It will also be investigated whether the tailored bundle of infection control measures has an influence on nosocomial infections (NI) and multi-resistant pathogens in the orthopedic and trauma surgery department, and whether this is economically detectable.

Furthermore, the development of a specific training program (Train the Trainer) for PLPs based on the data of a previously determined best practice approach in a pilot hospital should be conducted, with the aim of a better implementation of hygiene measures.

The employees' attitudes and knowledge of hygiene measures and other obstacles to adherence with hygiene measures before and after implementation of the bundle of measures will also be examined.

### 2.2 Study design

**Fig. 1:**
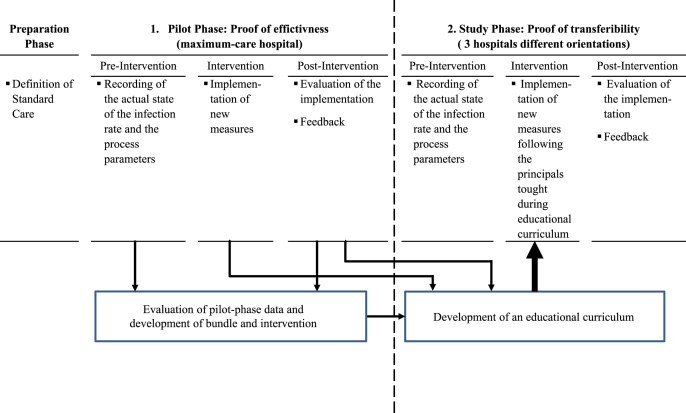
Study overview. The study consists of two study phases (pilot and study phases), each with pre-, intervention-, and post phases.

The multicenter prospective interventional pre-post cohort study is planned for a period of 3 years, divided into a pilot phase in one pilot hospital and a study phase in three additional study hospitals. The details of the study design are shown in Fig. 1.

### 2.3 Pilot phase

The pilot phase is divided into the preparation, pre-intervention, intervention, and post-intervention phases.

### 2.4 Pre-preparation phase (pilot)

In the pre-preparation phase, the following standard care is defined as the starting point for interventions in the pilot and study clinics:

Preoperative standards:

⁃ Inclusion of blood glucose and albumin in the laboratory profile
⁃ Hair removal using a clipper.

Intraoperative standards:

⁃ Single gloves for arthroscopy without implants
⁃ Double gloves for all other procedures
⁃ Glove change before implant insertion
⁃ Vancomycin soaking of transplant tendons in joint surgery
⁃ Gentamycin-containing cement for endoprosthesis implantation
⁃ No routine drainage in endoprosthetics
⁃ If drainage inserts removal is done <48 h post-surgery, no extension of antibiotic prophylaxis is required
⁃ Both seam and staple seam can be used

**Postoperative** standards:

⁃ Pre-/intraoperatively placed bladder catheter removed after <48 h
⁃ First dressing change after 48 h; short-term change only in the case of blood-drenched patches.

### 2.5 Pre-intervention phase (pilot)

#### 2.5.1 Outcomes for the primary endpoint

In the pre-intervention phase, the current actual states of the outcomes for the primary endpoint and process parameters are recorded. The primary endpoint is the occurrence of subsequent NI or death as a result of a nosocomial infection:

⁃ Postoperative wound infections
⁃ Thrombophlebitis
⁃ Urinary tract infections
⁃ Pneumonias
⁃ Primary sepsis
⁃ *C. difficile* infections

The following data are collected for the monitoring (surveillance) of these infections:

⁃ NI according to KISS and CDC definitions [26,27].
⁃ Microbiological, clinical, and radiological patient data.

The project team defined infection according to the German nosocomial infection surveillance systems (KISS) and the US centers for Disease Control and Prevention (CDC) definitions inclusive NHSN risk index. Diagnostically or therapeutically introduced foreign objects of non-human origin are defined as implants.

### 2.6 Process parameters

The process parameters should first determine where exactly in the specialist discipline a possible potential influence of the PLP on NI and SSI rates lies. Likewise, possible risk factors for the occurrence of NI or SSI are to be determined. In doing so, direct observations data (general hand hygiene adherence, hand hygiene during the dressing change process and process parameters during dressing change measurements, and hand hygiene adherence in the operating theater) of various activities and processes at the bedside and in the operating rooms will be reviewed for application of current recommendations.

The IPM that will be introduced in the intervention phase are aimed at reducing SSIs, as these are the most common NIs in the trauma surgery and orthopedic departments. Similarly, it is believed that by improving process parameters such as hand hygiene adherence rates and introducing decolonization measures for the patient, the number of other common NI in the hospital can also be reduced. For this reason, the infection rates of primary sepsis, thrombophlebitis, urinary tract infections, pneumonia, and *C. difficile* infections will also be considered.

**Possible obstacles to adherence with hygiene measures** (The survey will include nursing staff on the ward and in the operating theater as well as physicians)**:**

⁃ Employees' attitudes, knowledge, expectations, affects, and adherence of IPM
⁃ Subjective norm supervisor
⁃ Subjective norm department
⁃ Working environment.

#### 2.6.1 Intervention phase (pilot)

In the 3-month intervention phase, tailor-made hygiene measures (preoperative washing/decolonization, universal screening for MRSA/MSSA, correct antibiotic prophylaxis [30 min before incision with an adequate 1st generation dose of cephalosporin, adaptation to the body mass index and redosing if operation lasting more than 180 min], behavioral change in the operating theater, closed incision negative pressure wound Therapy [CiNPWT], standardized wound, and fixator care) are to be introduced in the pilot hospital.

The components of the infection prevention bundle to be implemented in the intervention phase were selected on the basis of a literature review [28].

#### 2.6.2 Post-intervention phase (pilot)

In the post-intervention phase of the pilot phase, the effectiveness of the measures is evaluated. For this purpose, the outcome parameters and the implementation of the process parameters are recorded. Furthermore, a training module for PLP will be developed to train their staff and introduce IPM in their departments. The concept developed in the pilot department will subsequently be transferred to three other study departments in the study phase.

### 2.7 Study phase

In the study phase, we examined the transmissibility of the infection prevention, implemented in the pilot phase, concept to three study hospitals of different sizes.

#### 2.7.1 Pre-intervention phase (study)

In the pre-intervention phase, the same outcome parameters are recorded as in the pre-intervention phase of the pilot phase.

#### 2.7.2 Intervention phase (study)

In the intervention phase of the study phase, the PLPs of the study hospitals are trained with the newly developed training concept in order to introduce hygiene measures in their departments and to train their staff. The next step is to analyze whether the PLP or his subsequent instructions of the staff has led to a reduction in NI by the implemented hygiene measures.

### 2.8 Post-intervention phase (study)

In the post-intervention phase, the realization of the newly introduced measures is evaluated, and first results are reported back to the employees.

**Fig. 2:**
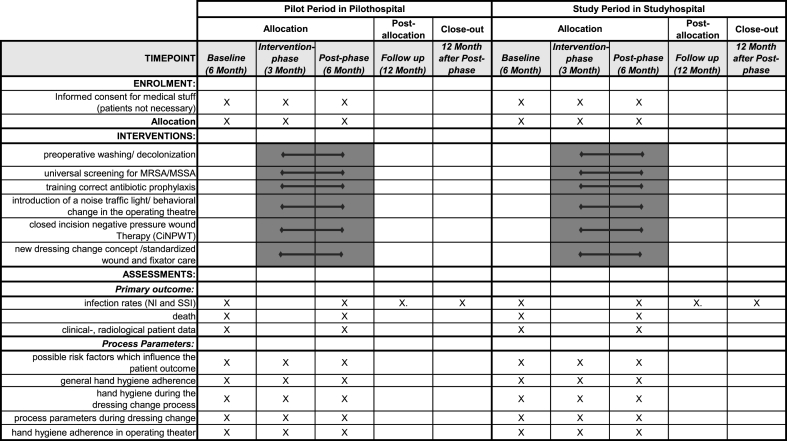
Spirit-Figure with allocations, interventions, and outcomes at different time points.

An overview of the outcome measurement at different time points is shown in Fig. 2.

### 2.9 Study participants

#### 2.9.1 Sample size calculation of number of to be included patients

In order to be able to measure sufficiently reliable results or statements about the effect of the intervention on infection rates and to generalize the results found, a classic a priori calculation of the required number of cases based on two patient cohorts and infection types was performed with G × Power, in which the required sample size was inferred by assuming the size of the difference or association (Fisher test for one-sided hypothesis [alternative: reduction in infection rate] at a ratio of 1:2 [pre-intervention: post-intervention]).

As a basis for calculation, the following assumptions were made during the pre-intervention phase: In the study, an overall infection rate for the orthopedics/trauma surgery department will be determined for the first time. Until now, there are only few reference values about an overall SSI infection rate but only infection rates for indicator surgeries like hip and knee endoprostheses. Since these infection rates do not reflect the reality on a standard trauma surgery ward and most postoperative wound infections are related to metal implants and their removal, an infection rate of 6.8% for metal removal or osteosynthesis material was used for the sample size calculation [29]. With an expected reduction of at least 50% for an infection rate of 3.4%, a power of 80% and a significance level alpha = 0.05, the number of patients required was: Pre-intervention n = 415, and post-intervention n = 830.

Next to postoperative wound infections, nosocomial urinary tract infections are the most frequent NI in Germany [2,30]. Since the interventions also aim to improve process parameters, such as increasing hand hygiene, it is assumed that the infection rate of nosocomial urinary tract infections will also be reduced.

We assume an infection rate in the pre-intervention phase of 4.42% for urinary tract infections from the reference data of the NRZ's ward KISS module for NI [31]. With an expected reduction of at least 50% for infection rate of 2.21%, a power of 80% and a significance level alpha = 0.05, the required number of patients was: pre-intervention n = 652, and post-intervention n = 1304.

This can be achieved in the two large hospital departments - each considered individually - and combining them increases the power.

By combining the study hospitals with a power of 80% and a reduction of only 25%, an infection rate of 3.3% can be achieved with the following case numbers:Pre-intervention n = 2954 and post-intervention n = 5908.

All wound infections, pneumonia and catheter-associated sepsis, and all other quantitatively relevant surgical interventions are also considered and evaluated descriptively.

## 3 Inclusion criteria for patients

We included patients based on the surgery for trauma surgery/orthopedic diagnosis and inpatient admission to one of the trauma surgery/orthopedic normal wards.

Infections are evaluated for the last surgery performed on a certain site.

If an infection occurs within 90 days of surgery, it is considered nosocomial according to the KISS definitions [26]. For consecutive procedures, the 90-day period begins after the last operation on this site, according to the KISS definitions for implants.

In case of polytraumas or other patients who temporary stay in the intensive care unit, only infections following the trauma surgery/orthopedics surgery (SSI) or which developed during their stay in the trauma surgery/orthopedics ward are considered. Infections will be considered to have developed in the normal ward if they appear more than 48 h after admission.

Only patients admitted to the normal ward were considered.

## 4 Exclusion criteria

Patients who leave the hospital prematurely against medical advice are excluded from the study.

SSIs which develop during a stay in an intensive care unit or less than 48 h afterwards will not be considered. Infections brought along, and infections acquired before the time of study were also excluded. Nosocomial acquired infections of the same type are only recorded once, even if they occur repeatedly. Exceptions are cases in which the infection occurs after a free clinical interval and a blocking period of 14 days from the beginning of the previous infection [26].

### 4.1 Economic outcome

The analysis of the routine DRG (Diagnosis Related Groups) depended on the hospital data and the infection surveillance data of the year 2016 in the pilot hospital. The interdisciplinary project team decided to include SSI, urinary tract infections, pneumonia, catheter sepsis, secondary sepsis, *C. difficile* infections, mortality, and antibiotic consumption as the outcome of NIs. The economic effects of NI prevention will be analyzed (i) from a hospital perspective and (ii) from a societal perspective. We used hospital routine accounting data to build our evaluation model. As direct variable costs (cost of materials used for treatment and cost of drugs) cannot be assigned per patient, the analysis focuses on indirect costs (i.e. opportunity costs of bed-days blocked by NI patients). Available routine data covers patient data (age, sex), ICD coding, OPS coding, data on ward capacity, DRG revenue per patient, and intervention costs.

To analyze the economic effects, different matching strategies are used. Based on the data available, a “core sample” for the evaluation will be established, covering all NI cases in the dataset. These NI cases are matched with patients without NI (normal cases) who were identical in the variable's main diagnosis, sex and age ( ±2 years). The average length of stay and the average DRG revenue per day will be calculated. In addition, linear regressions will be performed to analyze additional LOS.

Evaluating the data from a hospitals perspective, special emphasis will lay on the of (indirect) opportunity costs which play a major role for hospitals [32].

The opportunity costs are defined as the difference between Revenues Realized treating NI patients and Revenues Foregone for not using the bed capacity blocked by NI patients for the treatment of normal cases. The determinants of the bed-day opportunity costs are the length of stay of the NI patients, the daily revenues received for the normal cases, the occupation rate of the relevant hospital or department respectively, and the daily revenues received for NI patients.

Opportunity costs are calculated according to the following equation:

Opportunity Costs = Length of Stay NI x Daily Revenues Realized normal x Occupancy Rate – Length of Stay NI x Daily Revenues Realized NI.

If NIs prolong the average length of stay of the affected patients, the infection “blocks” beds, which are then no longer available for the treatment of “normal patients.” Thereby, the hospital loses revenue.

Evaluating the economic effects of NI prevention from societal perspective, additional LOS of NI patients becomes relevant. The corresponding data is valued with the productivity losses of NI patients affected.

In addition to the outcome parameters, the following literature-based packages of measures were defined for the intervention study.

### 4.2 Intervention measures

The structured development of an intervention bundle containing a complex intervention but no further details on team play and measure selection has been described so far. Therefore, a structured process for developing an intervention bundle is described here.

Schematic workflow of the intervention measures selection procedure:

1. Building an interdisciplinary team, which aims at reducing infection rates and organizing the selection process
2. Collecting basic information
a. Establishment of a local infection rate surveillance system
b. Systematic literature review
c. Process analysis of currently performed IPM and adherence
3. Compilation of a provisional, evidence-based infection prevention measure bundle based on the results of No. 2
4. Anticipation of needed economic resources (materials, infrastructure, staff) and human factors (reactance and possible counter measures) needed for the bundle
5. Modification of the provisional bundle taking into account the considerations in No. 4 to create a highly efficient, but at the same time feasible approach under local conditions
6 Assembling smaller work groups for detailed planning of implementation
7. Discussion of the work groups’ proposals and adoption by the interdisciplinary team
8 Implementation of the bundle

### 4.3 Outcome measures and data collection

#### 4.3.1 Data management and data safety

The data was routinely collected in accordance with HygmedVoNRW §8 and the Infection Protection law §23, and recorded in a newly created database [23,33]. An ethics vote was obtained for the conduct of the study and data collection. The same data was collected for pre- and post-observation.

In order to simplify data collection, some result parameters such as ASA scores, wound contamination class according to CDC, surgical urgency, preoperative antibiotic interval, and blood supply were recorded routinely.

## 5 Data sources

Data originates from clinical CCP, Ixserve, IMP, Hybase Statistics Program 2018 (epiNET AG) and recordings of the anesthesia department.

## 6 Data safety

The data collected in this non-experimental study are mainly routinely generated patient and process data, which are collected exclusively within the framework of current medical practice in accordance with HygmedVoNRW §8 and the Infection Protection law §23, and are protected and evaluated anonymously in accordance with the Federal Data Protection Act (BDSG) and the General Data Protection Regulation (GDPR) and according to §15 of the professional code of conduct for physicians of the Medical Association of North Rhine [23,33,34].

The analysis is performed anonymously in a separate database.

The data of the other test centers are encrypted and passed on via an external data carrier.

No written consent of the employees is required for open adherence analyses, as this is common infection prevention practice under the German Infection Protection Act, as is also the case with the “Clean Hands Campaign (ASH)” of the National Reference Center for Surveillance of 10.13039/100013765NI is supported by the German 10.13039/501100003107Federal Ministry of Health.

The employees involved are verbally informed before the start of adherence monitoring and their consent is obtained. The observations focus on the implemented interventions.

The employee surveys are carried out voluntarily and anonymously with prior clarification of content, purpose, voluntary participation, and the possibility of termination at any time without giving reasons.

## 7 Planned statistical analysis

Data from the different sources (laboratory data, anesthesia data, and surgical data) will be joined patient-wise into a single data table that allows for various analyses:

The risk of developing a nosocomial infection/surgical site infection will be modeled by a generalized linear model with logit link (logistic regression), eventually including random effects for the patient (if we measure patients more than once), staff and location [35,36]. Predictors in the model are pre- and post-phase, common covariates such as gender and age, as well as other variables such as the ASA score. Note that we may have to reduce the number of predictors if the number of NI is rather small. Given the model, we will generate well interpretable coefficients that describe the odds ratios for the predictors along with associated p-values calculated via Wald-type tests.

The adherence for hand hygiene and for various processes in the operating theater, and adherence for processing dressing changes is measured by checklists. Scores are derived from the checklists and modeled by linear mixed effect models or Tobit models [36,37] depending on the distribution of the residuals. Predictors in the models are pre and post-phase, staff level, and sex of staff members.

Pairwise comparisons of separate single items from hand hygiene, processes in the operating theater, and processes for dressing changes will be analyzed by Welch tests and permutations tests, if applicable.

Statistical calculations will be performed by the statistical software R and corresponding contributed packages coin, lme4, mgcv and VGAM [[37], [38], [39], [40], [41]].

## 8 Ethics approval and dissemination

The ethics vote (Application No 215/2017 of February 08, 2018) of the ethics commission of the University of Witten/Herdecke is informed about the following procedure and has agreed to it:

The data collected in this non-experimental study are mainly routinely generated patient and process data, which are collected exclusively within the framework of current medical practice in accordance with HygmedVoNRW §8 and the German Protection against Infection Act (§23 IfSG), and are protected and evaluated anonymously in accordance with the Federal Data Protection Act (BDSG) and the General Data Protection Regulation (GDPR) and according to §15 of the professional code of conduct for physicians of the Medical Association of North Rhine [23,33,34].

The compliance observation is an open observation and is only carried out on persons who agree with the observation. No written consent of the employees is required for the performance of open adherence analyses, as this is common infection prevention practice under the German Infection Protection Act, as is also the case with the ASH of the National Reference Center for Surveillance of 10.13039/100013765NI is supported by the German 10.13039/501100003107Federal Ministry of Health [21].

The employees involved were verbally informed before the start of adherence monitoring and their consent is obtained. The observations focus on the implemented interventions.

The employee surveys were carried out voluntarily and anonymously with prior clarification of content, purpose, voluntary participation, and the possibility of termination at any time without giving reasons.

First, the results of the respective study phases are reflected to the employees and flow into the development of the modular training program and internal further training. In addition, the results are presented at national and international congresses and published in leading professional journals.

## 9 Discussion

In the HygArzt study, we will investigate the possibilities for intervention and sustainable introduction as well as for the implementation of hygiene measures in orthopedics and trauma surgery by the PLP. The aim is to reduce the rates of NI with special regard to postoperative wound infections by successful implementation and the increased adherence with the IPM introduced and adjusted.

Routinely generated clinical, laboratory chemical and microbiological findings are used for processing analyses during patient care in order to optimize the nursing care and therapy of frequent clinical procedures in trauma surgery/orthopedics.

The “HygPFLEG” project (Development, implementation and evaluation of a modular Continuing education curricula for nursing staff responsible for hygiene by hygiene specialists) [42] has already shown that the implementation of hygiene measures by internal employees might be more successful. During this study a curriculum according to the train the trainer approach was developed that combined the contents of hospital hygiene with didactic and psychological methods. With this training program, the nursing staff was trained as hygiene nurses. It proved to be advantageous within the training program to implement hygiene measures together with the staff during every day clinical processes [29,43]. Based on the results of the “HygPFLEG” project, the staff at stations in the “HygArzt” study are also involved in the implementation of hygiene measures.

Despite precise planning, several limitations can occur at the level of the patients, the nursing, and medical staff as well as during the study.

On the one hand, an insufficient number of cases, patients, or observed employees could limit the study, so that the result parameters evaluated would have to be reduced in order to maintain the statistical quality criteria sufficiently.

A low adherence of employees with the interventions to be introduced could also be another limiting factor. However, these possible difficulties should be reduced by accompanying the intervention implementation, the adherence control as well as the direct behavioral feedback.

Likewise, the presence of an observer and the knowledge of the employees that are being observed could lead to the Hawthorne effect [[44], [45], [46]] and to social desirability, thus falsifying internal validity and results.

However, it is assumed that recurring observations lead to habituation and decrease the effect of social desirability.

The repeated observation of the small sample of nursing and medical employees could lead to distortions due to individual deviations in the form of under- or above-average adherence of individual employees.

In order to reduce possible distortions due to different assessment by the observers, standardized observation sheets are used, and the observers are trained in advance. During trial runs, the interrater reliability is checked in advance and the classification of the situation observations is reflected and discussed.

Possible effects on hygiene behavior due to the SARS-CoV-2 pandemic from March 2020 until the end of the study are taken into account in the evaluation of the observed data. The collected data should provide evidence that the action of a PLP could lead to a reduction in NI and adherence show the potential of this position. The PLP will be placed at the center of the implementation of hygienic prevention measures. Through the train the trainer approach, he himself becomes a trainer for his department. This approach is intended to achieve rapid implementation of preventive measures. In addition, a general best practice model will be developed, focusing on adherence-based hygiene measures in order to make them available to other hospitals or medical disciplines.

## 10 Current status of the project

The inclusion of patients started in March 2018 and ended in July 2020. Follow-up of included patients to see if they develop a postoperative wound infection will end in July 2021. The included departments treat mostly emergency patients. This number has not decreased despite SARS-CoV-2 pandemic situation. Only the number of elective patients might have decreased, as these surgeries were not performed during the acute phases of the pandemic.

Since the study is divided into a pilot phase and study phase, only the post-phase of the study phase is affected by the pandemic and the majority of the included patients were scheduled from the beginning purely emergency trauma surgery patients. For these two reasons, the pandemic number will not adversely affect the study results.

## 11 Consent for publication

Not applicable.

## 12 Availability of data and materials

Not applicable.

## 13 Ethics approval and dissemination

The ethics vote (Application No 215/2017 of February 08, 2018) of the ethics commission of the University of Witten/Herdecke is informed about the following procedure and has agreed to it:

The data collected in this non-experimental study are mainly routinely generated patient and process data, which are collected exclusively within the framework of current medical practice in accordance with HygmedVoNRW §8 and the German Protection against Infection Act (§23 IfSG), and are protected and evaluated anonymously in accordance with the Federal Data Protection Act (BDSG) and the General Data Protection Regulation (GDPR) and according to §15 of the professional code of conduct for physicians of the Medical Association of North Rhine.

The compliance observation is an open observation and is only carried out on persons who agree with the observation. No written consent of the employees is required for the performance of open adherence analyses, as this is common infection prevention practice under the German Infection Protection Act, as is also the case with the ASH of the National Reference Center for Surveillance of 10.13039/100013765NI is supported by the German 10.13039/501100003107Federal Ministry of Health.

The employees involved were verbally informed before the start of adherence monitoring and their consent is obtained. The observations focus on the implemented interventions.

## Funding

This work was supported by the 10.13039/501100003107Federal Ministry of Health Germany grant number (ZMVI1-2516FSB111). The 10.13039/501100003107Federal Ministry of Health Germany has exclusively provided funding for personnel and material resources for the research project. The funding is a state sponsorship.

## Authors' contributions

MN is the corresponding author, elaborated this article and developed tools for data acquisition of the study data and will collect them.

BM conducted an extensive literature research to prepare a literature review on which the selection of the infection prevention measure for the study bundle was based.

CK helped to design the study.

DB analyzed the patient data for economic outcome parameters.

DS developed the economic outcome parameters of the study.

SW conducted the sample size calculation and will support the statistical analysis of the study results.

UW conducted the sample size calculation and will support the statistical analysis of the study results.

FM created and designed the study, critically revised this manuscript and is scientific supervisor. FM and RO have the same contribution as scientific supervisor.

RO created and designed the study, critically revised this manuscript and is scientific supervisor and he is the head of the study. RO and FM have the same contribution as scientific supervisor.

All authors read and approved the final manuscript.

## Declaration of competing interest

The authors declare that they have no competing interests.
